# Mini-Review on the Harlequin Syndrome—A Rare Dysautonomic Manifestation Requiring Attention

**DOI:** 10.3390/medicina58070938

**Published:** 2022-07-15

**Authors:** Ioannis Mavroudis, Ioana-Miruna Balmus, Alin Ciobica, Alina-Costina Luca, Rumana Chowdhury, Alin-Constantin Iordache, Dragos Lucian Gorgan, Iulian Radu

**Affiliations:** 1 Department of Neuroscience, Leeds Teaching Hospitals, NHS Trust, Leeds LS2 9JT, UK; i.mavroudis@nhs.net (I.M.); rumana.chowdhury@nhs.net (R.C.); 2 Department of Exact Sciences and Natural Sciences, Institute of Interdisciplinary Research, “Alexandru Ioan Cuza” University of Iasi, Alexandru Lapusneanu Street, No. 26, 700057 Iasi, Romania; balmus.ioanamiruna@yahoo.com; 3 Department of Biology, Faculty of Biology, “Alexandru Ioan Cuza” University of Iasi, 700506 Iasi, Romania; alin.ciobica@uaic.ro (A.C.); lucian.gorgan@uaic.ro (D.L.G.); 4 Academy of Romanian Scientists, Splaiul Independentei Nr. 54, Sector 5, 050094 Bucuresti, Romania; 5 Center of Biomedical Research, Romanian Academy, B dul Carol I, No 8, 700505 Iasi, Romania; 6 Faculty of Medicine, “Grigore T. Popa” University of Medicine and Pharmacy, 16 Universitatii Street, 700115 Iasi, Romania; raduiuli@gmail.com

**Keywords:** Harlequin syndrome, autonomic nervous system impairment, unilateral, face flushing, sweating, anhidrosis, neurovascular, sympathetic, idiopathic, iatrogenic

## Abstract

Harlequin syndrome (HS) is a rare autonomic disorder. The causes and risk factors of the disease are not fully understood. Some cases of HS are associated with traumatic injuries, tumors, or vascular impairments of the head. Symptoms of HS can also occur in some autoimmune disorders, ophthalmic disorders, sleep disorders, and with certain organic lesions. In this context, a thorough review of the pathophysiology of HS in relation to neurological, ophthalmological, and dermatological conditions is necessary. In this mini-review, we aim to review the pathophysiological changes and underlying mechanisms in primary and secondary HS. Additionally, we discuss possible management approaches for patients with HS in light of the discussed pathological mechanisms. The main symptoms of HS that are correlated with autonomic nervous system impairments include sudden unilateral flushing of the face, neck, chest, and rarely arm, with concurrent contralateral anhidrosis. Despite reported co-occurring syndromes (such as cluster headaches), several studies have shown that HS could frequently overlap with other syndromes that are disruptive to the idiopathic nerve pathways. HS usually does not require any medical treatment. In some severe cases, symptomatic treatments could be needed. However, total symptomatic relief may not be achieved in many cases of HS. We therefore suggest an approach to comprehensive management of HS, which may lead to better long-term control of HS.

## 1. Introduction

Harlequin syndrome (HS) is a rare autonomic disorder of which its exact occurrence is unknown [1]. Due to its symptoms, the syndrome received its name from the typical red and black mask of the European fictional folklore character, the harlequin [1,2]. Whilst it can be diagnosed in newborns, HS does not typically exhibit genetic causes and many cases occur as a result of neurological impairment [3]. The main cause of HS is disruption of the sympathetic pathways, which could be exacerbated by stress, heat, and exercise [3]. However, some HS cases are associated with traumatic, tumorous, or vascular impairments of the head, autoimmune disorders, eyes, or sleep disorders [4,5]. Thus, HS could also be secondary to organic lesions or due to iatrogenic causes. In this context, further neurological, ophthalmological, and dermatological investigations are needed to describe the correlation between causes and effects.

In this mini-review, we aimed to focus on comparing the pathophysiological traits and mechanisms in primary and secondary HS cases. We also aimed to discuss possible management approaches with relation to the discussed pathophysiology.

## 2. Materials and Methods

A literature search was conducted on the main scientific databases (PubMed/Medline, Google Scholar, Embase, and ScienceDirect) using keywords, such as: “Harlequin syndrome”, “unilateral”, “face flushing”, “sweating”, “anhidrosis”, “autonomic nervous system impairment”, “physiopathology”, “neurovascular”, “sympathetic”, “idiopathic”, “iatrogenic”, “diagnosis”, “treatment”, and combinations. Papers written in the English language including research articles, case reports, reviews, editorials, comments, and opinions were considered. The selection process was conducted by two separate researchers, and the common consensus of the authors settled all differences in opinion. The initial search consisted of 22 relevant studies, 18 of which described adult cases and 4 of which described infant cases. Following criteria-based sorting, no study was excluded.

## 3. Symptomatology and Pathological Mechanisms

The main HS symptomatology is correlated with autonomic nervous system impairment, which leads to sudden unilateral flushing of the face, neck, chest, and arm, concurrent with contralateral anhidrosis. The occurrence of HS symptoms is age-independent, but usually reported as exacerbated by stress, emotional reactions, exercise, heat, and spicy foods [1,5].

While co-occurring syndromes, such as cluster headaches, were reported, several studies showed that HS symptoms could frequently overlap with other syndromes disruptive to the idiopathic nerve pathways (Horner syndrome, Ross syndrome, and/or Adie syndrome) [5]. In this way, it was showed that up to 64% of HS patients exhibit an ipsilateral Horner syndrome’s abnormal pupil, while 13% of patients could show areflexive tonic pupils. Moreover, 4% of HS patients showed overlapping tonic pupils and Horner syndrome [6].

Interestingly, several isolated reports suggested a possible congenital cause of HS. Vidal Esteban et al. [7] suggested that congenital HS could occur in less than 6% cases and only concomitant to Horner’s syndrome. Both Vidas Esteban et al. [7] and Elnahry et al. [8] reported concomitant congenital HS and Horner’s syndrome in infants. However, Elnahry et al. [8] suggested that forceps- or vacuum-assisted deliveries could be a possible cause of HS, thus indicating an iatrogenic rather than congenital cause. However, HS was also reported in children with an unremarkable birth history and uneventful medical history (as was the case of a 6-year-old South Korean boy) [9]. On the other hand, Ritter Hans-Bitner et al. [10] recently reported apparently idiopathic HS in a 34-year-old man with anxious personality disorder under observation. The correlation between the autonomic nervous system and psychiatric disorders was previously demonstrated in many neuropathological impairments, such as affective disorders, schizophrenia, epilepsy, and autism [11,12,13,14].

Regarding the physiology of sensorimotor sympathetic innervation, the first neurons are located centrally in the hypothalamus, whereas the second neurons reside in the mediolateral or lateral spinal cord columns, the presynaptic pathway connecting the anterior roots and T1-L2 anterior rami innervating the trunk and abdominopelvic viscera [15]. By contrast, the sympathetic innervation from head to thorax and upper limbs is supplied by the postganglionic fibers of the spinal nerves C2–C8 [16,17].

The cervical sympathetic chain has three adjacent ganglia—the superior cervical, the middle and the inferior cervical ganglia—which in 80% is combined with the first thoracic ganglion, and forms the cervicothoracic ganglion, or stellate ganglion [18]. Blood supply of the sympathetic trunk is delivered by ascending cervical and superior thyroid arteries, the inferior thyroid artery and the subclavian arteries [18].

Considering the physiology of the face and neck innervation, it was described that the sudomotor and vasomotor innervations travel from the T2–T3 ventral spinal roots to the superior cervical ganglion through the sympathetic chain [19]. Moreover, the postganglionic fibers innervating the forehead and nose are proximal to the carotid artery, while the other facial areas’ postganglionic neuron fibers are also in the proximity of the external carotid artery [19]. It could be suggested that HS sudorific and vascular responses could be the result of insults to the neck-located carotid artery surroundings. Similarly, the efficiency of stellate ganglion block in HS symptom alleviation could be supported by arm innervation physiology, as the preganglionic fibers innervating the arms connect the stellate ganglion with the T4 spinal area [19].

## 4. Etiopathology

Regarding causes of HS, approximately 54.6% of reported cases have idiopathic etiology, while in 45.4% of cases, the HS-associated symptomatology occurred secondarily, concurring with other autonomic nervous system impairments, or iatrogenically (Table 1). The secondary causes of HS include trauma, stroke, autoimmune conditions such as multiple sclerosis, hyperthyroidism, viral infections, and spinal cord syrinx [5,20,21].

The iatrogenic etiology of HS was previously reported by Fringeli et al. [22] in a non-sensorimotor impaired 55-year-old female patient with large median and right paramedian disc hernia-caused right C7 radiculopathy following C6–C7 fusion discectomy and additional anterior spondylodesis. In this case, the patient immediately developed visible HS-suggestive symptoms on the right side of the face (flushing and excessive sweating), and contralateral facial anhidrosis, miosis, and ptosis, as described in the initial report [2]. The cervical spine CT angiographies revealed no damage, despite the drain being proximal to the sympathetic trunk and the left common carotid artery posterior wall [22]. Thus, blood flow interruptions or partial/total temporary artery cannulation and sympathetic nerve damage could also lead to HS symptomatology development.

Supporting this hypothesis, Coleman and Goddard reported that HS occurred after internal jugular vein catheterization in a 46-year-old woman who underwent laparotomy and right lumbar intraabdominal intestinal contents’ drainage following a transduodenal sphincterotomy [23]. Similarly, Sarikaya et al. [24] described HS-associated symptoms in a 52-year-old patient that underwent spontaneous left cervical carotid artery dissection. In this context, it was shown that the stellate—superior cervical ganglion—sympathetic chain neurovascular compression was caused by the elongated inferior thyroid artery and led to HS symptoms development in a 55-year-old female patient [19]. HS was also reported as a possible consequence to neck Schwannoma excision in a pediatric patient [25]. A recent case report of a healthy 52-year-old man with sudden onset HS also suggested Schwannomas in the cervical chain ganglion as a possible secondary cause of HS [26].

HS occurrence was also associated with post-operative malignancies. Sullivan et al. reported HS symptoms occurring in a female patient following T3 erector spinae plane block for radical mastectomy and axillary dissection [27]. Burlacu and Buggy described coexisting HS and Horner syndrome following left mastectomy and immediate latissimus dorsi reconstruction in a 55-year-old female patient receiving upper thoracic paravertebral anesthesia [28]. Similarly, right axillary and supraclavicular lymph node enlargement 18 years following squamous non-small cell lung cancer treated by surgery and chemotherapy was reported to determine the occurrence of HS symptoms in a 72-year-old man [3]. In this case, CT scans showed that enlarged and hypodense axillary and supraclavicular lymph nodes could exhibit compressive actions on the carotid artery, the internal jugular vein, and subclavian artery in the proximity of which they are located [3,29].

Moreover, Bremner et al. [6] reported that 23% of 39 serial HS patients filled the criteria for multiple HS causes, including forceps delivery-caused brachial plexopathies, pure autonomic failure/small fiber neuropathy, multiple-system atrophy, Guillain–Barré syndrome, unspecified dysautonomia, surgically and radiotherapy-treated Pancoast lung tumor (poorly differentiated adenocarcinoma), thoracic sympathectomy, and non-neuropathic type 1 diabetes. Additionally, the report showed that multiple system atrophy and pure dysautonomia could be associated with HS in the long term [6].

However, some other independent disorders, such as lupus erythematosus, trigeminal neuralgia, and idiopathic bladder dysfunction (associated with unidentified lower spinal cord lesion, previous axillary surgery for hyperhidrosis and vasovagal episodes), could also exhibit HS-related symptoms [6].

Pediatric iatrogenic HS was also described following elective neck dissection for a left cervical lymphatic malformation [30], and elective resection of a neck mass [31].

Another possible iatrogenic cause of HS was described by Van Slycke et al. [32] in a 74-year-old woman following compressive retrosternal goiter thyroidectomy. Emotional changes, physical effort, or heat-related recurring sudden left-sided facial flushing and sweating were reported post-operation. In some cases, HS could fully remit with no further relapses [32].

## 5. Management and Treatment of Harlequin Syndrome

For proper management and treatment, the exact diagnosis of HS symptoms and cause are required. Detailed history and clinical examination, autonomic testing, and imaging could exclude HS secondary causes. Clinical examination should include a detailed neurological evaluation, examination of the thyroid, and search for pathology around the thoracic sympathetic outflow [29,33]. Work-up of HS should include a CT or MRI of the brain and cervicothalamic spine including the area of the thoracic sympathetic chain to exclude space-occupying lesions, infarction, or other etiologies [34]. Sweating tests, cardiovascular reflex tests to recognize autonomic failure, microneurography from the peroneal nerve to record muscle sympathetic nerve activity and skin sympathetic nerve activity are also valuable [35,36]. A skin biopsy may also be helpful to detect small fiber neuropathy.

HS does not usually require any medical treatment, but there are several symptomatic treatments available (Table 2). In severe cases, ipsilateral surgical sympathectomy could prevent compensatory flushing and sweating. Due to its sympathetic nervous relief and vasodilatory effect, stellate ganglion blocking could also be repeatedly administered in HS patients. HS-related excessive sweating could also be alleviated by botulinum toxin treatment, which safely blocks acetylcholinergic signaling to sweat glands [37,38,39].

## 6. Conclusions

Harlequin syndrome is a rare neurological disorder affecting the face and neck autonomic nervous system due to idiopathic and iatrogenic causes, affecting both adults and children. Nerve lesions or vascular cannulation during surgical procedures are most commonly followed by HS symptoms. In many cases, the symptoms remit with no treatment; however, for those with persistent HS symptoms, a detailed assessment of the cause and extent of autonomic dysfunction may aid long-term patient management and quality of life.

## Figures and Tables

**Table 1:** Most common causes of secondary Harlequin syndrome (as summarized by all the described cases).

Organic/Structural Causes	Iatrogenic Causes
Cervical syrinx	Paravertebral thoracic block
Intermedullary astrocytoma	Jugular vein catheterization
Stroke/infract, Diabetic neuropathy	Neck mass resection, including thyroid goiter
Thoracic neurofibroma	Thoracic sympathectomy
Pancoast tumor	Neuropraxia
Compression of sympathetic chain by elongated thyroid artery	Pharmacological causes
Small fiber neuropathy/Pure autonomic failure/Unspecified dysautonomia	Cage fusion and additional anterior interbody spondylodesis for cervical radiculopathy
Carotid artery dissection	**Possible causes**
Mediastinal neurinoma	Discoid lupus erythematosus
Guillain–Barré syndrome	Trigeminal neuralgia
Brachial plexopathy, Multiple system atrophy	Idiopathic bladder dysfunction (unidentified lower spinal cord lesion)

**Table 2:** Assessment and management of Harlequin syndrome.

History	History including past medical and family medical history; History of previous surgical procedures/operations;
Physical examination	Neurological examination including tendon reflexes and pupils reaction; Physical examination including thyroid and search for pathology around the thoracic sympathetic outflow;
Imaging	MRI brain and cervical-thalamic spine including the area of thoracic sympathetic chain, chest X-ray, and thyroid ultrasound;
Other investigations	Nerve conduction studies, cardiovascular reflex test, microneurography from peroneal nerve, skin biopsy;
Management	Surgical sympathectomy ipsilateral to the affected side, stellate ganglion block, Botulinum toxin.

## Data Availability

Not applicable.
